# Repeated Freezing Procedures Preserve Structural and Functional Properties of Amniotic Membrane for Application in Ophthalmology

**DOI:** 10.3390/ijms21114029

**Published:** 2020-06-04

**Authors:** Olena Pogozhykh, Nicola Hofmann, Oleksandr Gryshkov, Constantin von Kaisenberg, Marc Mueller, Birgit Glasmacher, Denys Pogozhykh, Martin Börgel, Rainer Blasczyk, Constança Figueiredo

**Affiliations:** 1 Institute of Transfusion Medicine and Transplant Engineering, Hannover Medical School, 30625 Hannover, Germany; Pogozhykh.Denys@mh-hannover.de (D.P.); Blasczyk.Rainer@mh-hannover.de (R.B.); 2 German Society for Tissue Transplantation (DGFG), 30625 Hannover, Germany; Nicola.Hofmann@gewebenetzwerk.de; 3 Institute for Multiphase Processes, Gottfried Wilhelm Leibniz Universität Hannover, 30167 Hannover, Germany; Gryshkov@imp.uni-hannover.de (O.G.); Mueller@imp.uni-hannover.de (M.M.); Glasmacher@imp.uni-hannover.de (B.G.); 4 Department of Obstetrics and Gynecology, Hannover Medical School, 30625 Hannover, Germany; vonKaisenberg.Constantin@mh-hannover.de

**Keywords:** amniotic membrane, AmnioClip-plus, cryopreservation, ocular surface disorder, ultrastructure, growth factors, cryomicroscopy

## Abstract

For decades, the unique regenerative properties of the human amniotic membrane (hAM) have been successfully utilized in ophthalmology. As a directly applied biomaterial, the hAM should be available in a ready to use manner in clinical settings. However, an extended period of time is obligatory for performing quality and safety tests. Hence, the low temperature storage of the hAM is a virtually inevitable step in the chain from donor retrieval to patient application. At the same time, the impact of subzero temperatures carries an increased risk of irreversible alterations of the structure and composition of biological objects. In the present study, we performed a comprehensive analysis of the hAM as a medicinal product; this is intended for a novel strategy of application in ophthalmology requiring a GMP production protocol including double freezing–thawing cycles. We compared clinically relevant parameters, such as levels of growth factors and extracellular matrix proteins content, morphology, ultrastructure and mechanical properties, before and after one and two freezing cycles. It was found that epidermal growth factor (EGF), transforming growth factor beta 1 (TGF-β1), hepatocyte growth factor (HGF), basic fibroblast growth factor (bFGF), hyaluronic acid, and laminin could be detected in all studied conditions without significant differences. Additionally, histological and ultrastructure analysis, as well as transparency and mechanical tests, demonstrated that properties of the hAM required to support therapeutic efficacy in ophthalmology are not impaired by dual freezing.

## 1. Introduction

The placenta is a temporary organ with a broad variety of unique physiological functions, as well as a distinctive structure and composition, which represent an immense potential for efficient application in the field of regenerative medicine [1,2,3,4,5,6,7,8,9]. The virtually unlimited availability of placental material along with highly pronounced therapeutic effects in a wide range of clinical cases provide support for the patients for over a century of successful clinical use and promote the further development of novel therapeutic strategies and approaches [3,8,9,10,11,12,13,14,15]. In the majority of countries, the application of placental components faces no or low ethical issues. Usually, women positively evaluate this opportunity and otherwise consider the material as "clinical waste" [8,9]. The donation of the placenta is physiologically harmless to the living donor, and it delivers large amounts of biological material suitable for application in the initial state or after processing [9,16]. 

In particular, the hAM yields distinctive efficiency in the field of ophthalmology during the treatment of various corneal pathologies and wound healing [1,2,3,4,5,6,8,9,17]. Its natural transparency, together with its mechanical strength and elasticity, provide visibility, support, and protection for the corneal epithelium, where AM serves as a biological dressing [4,9]. The capability to stimulate the proliferation and migration of limbal stem cells and the potential suppression of neo-angiogenesis, inflammation, and scarring, as well as reported antimicrobial action, are among the distinct properties of the hAM that allow for the achievement of long-term curative effects in the treatment of numerous ocular surface disorders, including limbal stem cell deficiency, conjunctive reconstruction and glaucoma surgeries, scleral melts and perforations [1,3,4,6,7,17,18,19]. Such growth factors as epidermal growth factor (EGF), transforming growth factor beta 1 (TGF-β1), hepatocyte growth factor (HGF), and basic fibroblast growth factor (bFGF), along with certain extracellular matrix proteins, like hyaluronic acid and laminin, are considered to be key players that contribute to the unique regenerative efficiency of the hAM [1,9,20,21,22]. Moreover, amniotic tissue possesses significantly downregulated to a virtual lack of expression human leukocyte antigens, which contributes to low immunogenicity after allogenic transplantation [23,24].

There are several strategies of the application of hAM to an eye of a patient. Conventional approaches use its fixation with sutures to the ocular surface [2,4]. However, surgical intervention to the site of inflammation and pathological processes entails further traumatization and wounding, and it may lead to bleeding, scarring, or other related adverse effects. At the same time, the most pronounced clinical effect of the hAM is observed mainly within the first week after transplantation [25]. This further complicates the use of surgical administration, since it is potentially required on a weekly basis. To avoid invasive surgery, one of the alternative approaches is based on the fixation of the hAM to the ocular surface with fibrin glue [26,27,28]. However, there are reports of negative effects and disadvantages of such an application [27,28,29]. Recently, a novel strategy was developed to eliminate the aforementioned complications of surgical fixation and fibrin glue application. This approach is based on a ring system (AmnioClip), where the hAM is mounted between two rings (the so called AmnioClip-plus). Such a system can be easily introduced to a patient like a contact lens without surgical intervention and can be replaced on a regular basis when required [2]. 

Independently of the choice of the transplantation strategy of the hAM into the eye, current clinical application is virtually always preceded by cryopreservation steps [3,9]. Cryopreservation is not only inevitable due to the necessity to have time to perform a range of obligatory safety tests of the donor material—it also permits the storage, transportation, and delivery of the hAM to the clinical setting in an integral and ready-to-use manner [1,10,30]. There are numerous approaches for the cryopreservation of hAM, including the application of glycerol, dimethyl sulfoxide, and trehalose as cryoprotective agents (CPAs), as well as direct freezing without CPAs (fresh frozen) [2,9,30,31,32,33,34]. Additionally, lyophilization (freeze-drying) or air-drying methods can be applied for the storage and clinical logistics; however, the latter approach has proven to be less efficient than classical cryopreservation [35,36,37,38,39]. 

While at least one freezing cycle is mostly present in the chain from retrieval of the hAM from the donor until the delivery to a patient, realities in clinical practice often presume the introduction of a second freezing step. The necessity for a second freezing may emerge due to a range of handling procedures due to logistical reasons and the peculiarities of application processes [2,34,40,41]. The freezing procedure, as well as temperature fluctuations and freezing cycles, may alter the integrity and properties of biological objects [10,33,37,40,42]. Therefore, this study was aimed at the comprehensive characterization of the clinically relevant parameters of the hAM before cryopreservation, as well as after one and two cycles of freezing without CPAs. We investigated the content of growth factors and extracellular matrix proteins in hAM lysates and their release in a conditioned medium, and we also analyzed possible alterations in transparency, mechanical properties, morphology, and ultrastructure. 

## 2. Results

### 2.1. Evaluation of Content and Release Potential of Growth Factors and Extracellular Matrix Proteins of hAM 

The contents of growth factors and extracellular matrix proteins present in the hAM was quantified in hAM lysates. The potential of the hAM to release these substances was evaluated by quantification in a conditioned medium after 24 hours of incubation. The values were normalized to the total protein amount in the lysates or in a conditioned medium. 

**Figure 1:**
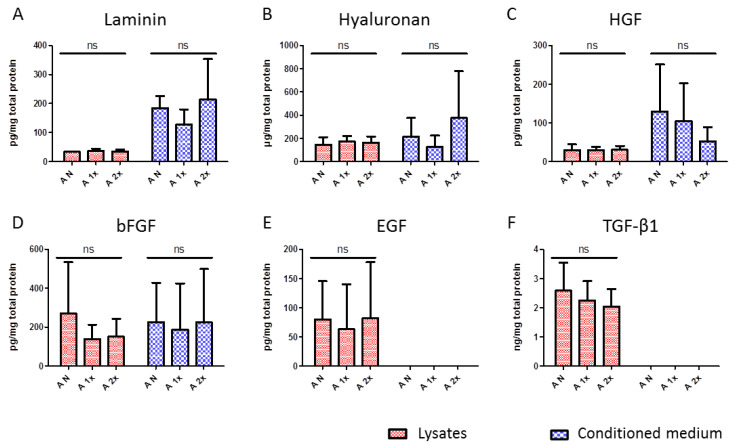
Content of growth factors and extracellular matrix proteins in lysates and the conditioned medium from non-frozen (A N), one-time frozen (A 1×), and two times frozen (A 2×) human amniotic membrane (hAM). Histograms show results of ELISA analysis for the presence and quantity of (**A**)—laminin; (**B**)—hyaluronic acid; (**C**)—hepatocyte growth factor (HGF); (**D**)—basic fibroblast growth factor (bFGF); (**E**)—epidermal growth factor (EGF); and (**F**)—transforming growth factor beta 1 (TGF-β1) in hAM lysates directly after lysis, as well as in the conditioned medium incubated with hAMs for 24 hours. Results are displayed as relative to the total protein amount in each sample (*n* = 5). The data are presented as mean ± SD; ns—non-significant difference (*p* > 0.05).

Lysates from non-frozen hAM samples contained 33.8 ± 1.9 pg/mg of laminin and did not significantly differ from the laminin content in lysates from the single frozen and twice frozen hAM samples, being 36.9 ± 7.7 and 35.6 ± 6 pg/mg, respectively (Figure 1A). Besides, no significant difference was observed in the laminin content released to the conditioned medium, being 184.97 ± 40.5 pg/mg released from the non-frozen hAM samples, 187.89 ± 52.3 pg/mg from the single frozen samples, and 214.3 ± 138.6 pg/mg from the frozen twice samples. More pronounced variations were observed between the different donors than between the different temperature modes. 

Hyaluronic acid was present in a concentration of 147.6 ± 62.7 µg/mg in the lysates from the non-frozen hAM, 173.4 ± 48.2 µg/mg from the single frozen hAM, and 163.3 ± 53.8 µg/mg from the double frozen hAM (Figure 1B). No significant differences were observed between the samples subjected to the studied temperature modes. The conditioned medium contained 214.2 ± 161.7 µg/mg of hyaluronic acid released by the non-frozen hAM, as well as 127.9 ± 95.6 and 375.1 ± 402.8 µg/mg from the hAM frozen once and twice, respectively. The conditioned medium possessed especially pronounced inter-donor differences in the capability to release hyaluronic acid. Standard deviations between the donors prevailed in the possible differences that could be caused by temperature modes (Figure 1B). At the same time, within a single donor, no significant differences were observed in relation to the temperature modes (data not shown). 

The content of HGF was stable in the hAM lysates independently of the temperature modes, being 29.8 ± 14.5 pg/mg in the non-frozen samples, 29.5 ± 8.7 pg/mg in single time frozen samples, and 30.6 ± 10.2 pg/mg in two times frozen samples (Figure 1C). The conditioned medium contained 129.6 ± 120.9 pg/mg of the relative HGF amount released from the non-frozen hAM, 104.9 ± 97.3 pg/mg from the once frozen hAM, and 52.9 ± 36.1 pg/mg from the twice frozen hAM. Again, hAMs from different donors possessed varied capabilities of this growth factor release, which resulted in high standard deviations between the donors. 

Interestingly, while the amounts of laminin, hyaluronic acid, and HGF were consistent in the hAMs of different donors and did not change during the repeated impact of subzero temperatures, the content of bFGF was distributed unevenly between the donors, as observed in the lysates (Figure 1D). Lysates from the non-frozen hAM contained 271.1 ± 262.8 pg/mg of bFGF, and lysates from the single frozen and two times frozen hAMs contained 138.3 ± 73.8 and 150.8 ± 91.9 pg/mg, respectively. The non-frozen hAM released 225.8 ± 201.7 pg/mg of bFGF to the conditioned medium, and the cases of single and double freezing hAMs released 187 ± 237.8 and 224.2 ± 274.5 pg/mg, respectively. Here as well, interdonor distribution was exceedingly uneven.

Lysates from the non-frozen hAM contained 80.1 ± 65.5 pg/mg of EGF, along with 63.3 ± 76.3 pg/mg after one cycle of freezing and 81.8 ± 96.1 pg/mg after two cycles, with no significant differences between the temperature modes and with pronounced variations between the hAMs of different donors (Figure 1E). Interestingly, no traces of EGF were detected in the conditioned medium after 24 hours of incubation. 

Additionally, no significant differences were observed between the content of TGF-β1 in the lysates from the non-frozen hAM and the hAM that was frozen once and twice, at 2.6 ± 0.9, 2.2 ± 0.6, and 2.0 ± 0.6 ng/mg, respectively (Figure 1F). Similarly to EGF, TGF-β1 was not observed to be released to the conditioned medium at the studied time point in any of the samples. 

In general, it was found that hAM contained growth factors EGF, TGF-β1, HGF, and bFGF, as well as the extracellular matrix proteins hyaluronic acid and laminin, in amounts that varied from donor to donor but were not significantly altered by single or double freezing procedures. At the same time, while HGF, bFGF, hyaluronic acid, and laminin were found in the conditioned medium incubated with the non-frozen and frozen hAMs, no traces of released EGF and TGF-β1 were observed in any of the samples after 24 hours of incubation. 

### 2.2. Evaluation of Morphological and Ultrastructural Changes in hAM after Multiple Freeze–Thaw Cycles

#### 2.2.1. Histological Assessment of hAM Morphology with HE Staining

**Figure 2:**
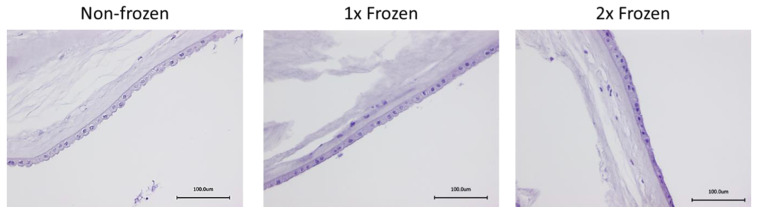
Representative histology images of the non-frozen, one-time frozen (1×) and two times frozen (2×) hAM. Hematoxylin and eosin (HE) staining displayed similar morphology of the hAM after all studied temperature modes. Scale bar 100 µm.

The evaluation of the structural morphology of the single and double frozen hAMs with the non-frozen samples was performed by hematoxylin and eosin (HE) staining. The HE staining allowed us to distinguish cellular components via the staining of cellular nuclei with hematoxylin and the extracellular matrix via staining with eosin. A microscopic analysis showed no visually significant evidence of the hAM tissue disruption of cryopreserved samples in comparison to the non-frozen samples and displayed intact characteristic structural components of the hAM, such as an epithelial layer, a basement membrane, and a stromal matrix (Figure 2). The morphology of the double frozen hAM samples was visually similar to those frozen one time. 

#### 2.2.2. Analysis of Ultrastructure of hAM with SEM

**Figure 3:**
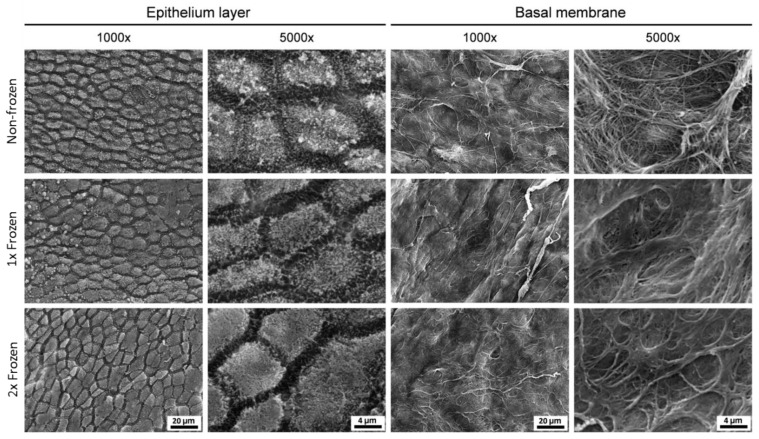
Representative SEM images of the non-frozen, one-time frozen (1×), and two times frozen (2×) hAM. The images at 1000× and 5000× magnification show the epithelium layer and the basal membrane. Scale bars are 20 and 4 µm for 1000× and 5000× magnifications, respectively.

In addition to histology, a deeper ultrastructure analysis of possible morphological changes in the hAM that may occur due to freezing procedures was performed by SEM (Figure 3). No noticeable differences were revealed in the appearance of the epithelium layer of the one time frozen membrane in comparison to the non-frozen membrane. However, a slight disintegration of cell–cell contact was observed in this layer after the second freeze–thaw cycle at a higher magnification of 5000×. At the same time, the structural morphology of the basal membrane remained intact after two freeze–thaw cycles, and, thus, the capability to provide mechanical stability for the application in ophthalmology was retained.

#### 2.2.3. Cryomicroscopic Analysis of hAM Behavior at Subzero Temperatures

**Figure 4:**
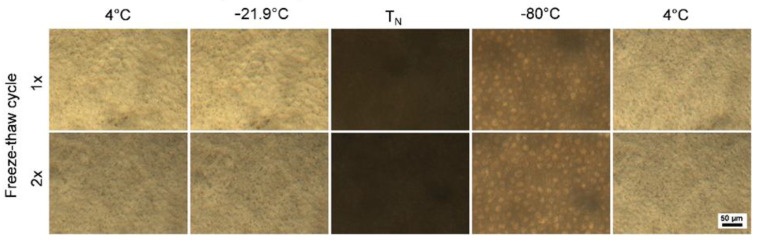
Cryomicroscopic images of the hAM during the first (1×) and second (2×) freeze–thaw cycles. The images represent the appearance of the membrane before freezing (left: 4 °C), directly before ice formation (−21.9 °C), after ice formation (T_N_), at the aimed storage temperature (−80 °C), and after thawing (right 4 °C). Scale bar 50 µm.

Cryomicroscopic analysis was performed to track the behavior of the hAM during the freezing and thawing procedures at the first and the second freeze–thaw cycles. No visible alterations or morphological and structural disintegrations were observed in the samples, regardless of the number of analyzed temperature cycles (Figure 4). The ice formation during the first and the second freeze–thaw cycles occurred at the temperatures of nucleation (T_N_) of −21.3 ± 0.6 and −19.5 ± 0.8 °C, respectively.

### 2.3. Assessment of Mechanical Properties of hAM

**Figure 5:**
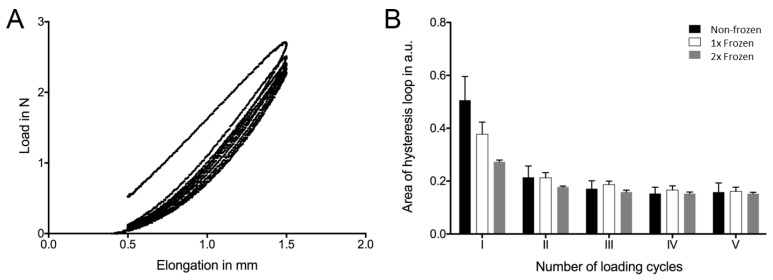
Assessment of the mechanical properties of the non-frozen, one-time frozen (1×), and two times frozen (2×) hAM. Representative hysteresis diagram of the hAM sample (**A**). The area of the hysteresis loop decreased with the increasing number of loading and unloading cycles. The biomechanical behavior was observed for all samples regardless of the number of freezing and thawing procedures (**B**). The difference between the first and second loading cycle decreased with the increasing number of freezing and thawing procedures (*n* = 3).

Mechanical testing was performed to investigate the influence of the freeze and thaw procedures on the biomechanical properties of the hAM. Hysteresis loops show the typical mechanical behavior of biological tissues (Figure 5A). The size of the loop or enclosed area was largest in the first cycle and decreased the most between the first and second cycle, followed by a further minimal decrease with each cycle (Figure 5B). This behavior occurred regardless of the number of freeze–thaw treatments. Absolute values differed, showing major differences in the first cycle. From the second cycle, the differences between the groups decreased. 

### 2.4. Transparency

**Figure 6:**
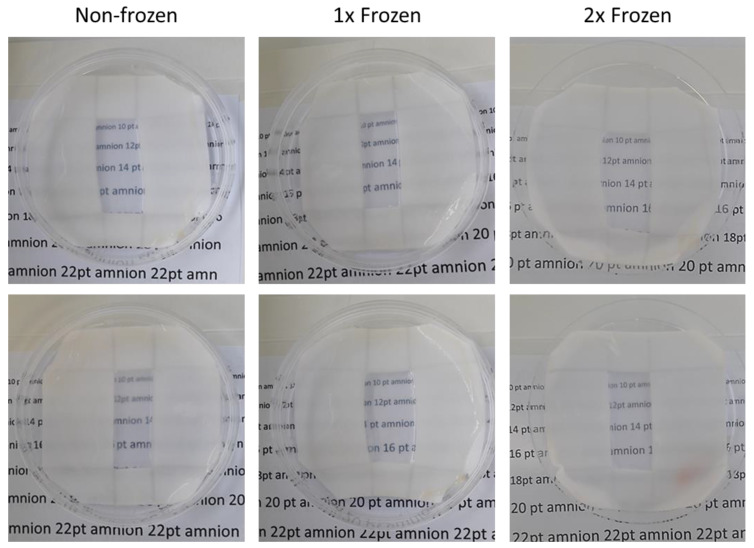
Transparency test. The images display the visibility of a printed text of various font sizes through non-frozen, one-time frozen (1×), and two times frozen (2×) hAMs placed on a carrier with an aperture of 15 × 30 mm over the text in a 100 mm cell culture dish.

Since transparency is among the key physical parameters required from the hAM for application in ophthalmology, whether it could be impaired with the impact of repeated freezing and thawing was tested. All studied non-frozen hAMs were equally transparent upon preparation, with a clear possibility to read the text placed underneath the membrane (Figure 6). Neither the single nor double freezing–thawing of hAM caused a considerable effect to this parameter, with the clear visibility of the text remained unaltered. 

## 3. Discussion

Nearly unlimited availability, in combination with a whole range of unique natural features and peculiar properties—along with decades of successful clinical application and comprehensive research—determine the invaluable potential of placental material to significantly contribute to the rapidly evolving field of regenerative medicine [8,9,43]. Mesenchymal cells of placental origin, umbilical cord blood, umbilical cord tissue, placental extracts and tissue fragments, amniotic and chorionic membranes provide a solid material basis for clinical studies and clinical application, as well as facilitate progress in bioengineering and fundamental biomedical science [9,16,44,45,46]. Among this wide range of possibilities of the utilization of placenta and placental derivatives, the hAM is among the most being extensively used in clinics [1,2,4,6,7,19,47,48]. Exceptionally distinct clinical effects achieved by using the hAM have been described in the treatment of ocular surface pathologies [1,2,3,4,6,17,25,36]. First publications on clinical use of hAM dating back to the works of J. Davis in 1910 and the first application in ophthalmology published in 1940 served as a basis for further rapid progress in the field with thousands of treated patients being reported since the mid-20th century [5,8,11,49]. 

While direct applications of freshly retrieved hAM have been used in the past, modern requirements for clinical use require at least one step of cryopreservation [3,9]. Besides the regulations and necessity for the safety tests of donor material, cryopreservation allows for the accumulation, long-term storage, transportation, and delivery of the hAM to a clinical setting in a ready-to-use manner or for further processing. Here a second freezing–thawing step is frequently involved [2,34,40,41]. However, the phase transition of water from the liquid to the solid state and vice versa may result in significant structural and functional alterations of biological objects and biomacromolecules [10,33,37,40,42]. Therefore, in the frame of this study, we analyzed whether the double freezing–thawing procedure could alter the structural and functional properties of the hAM, which may be relevant to practical application in regenerative ophthalmology for the correction of ocular surface pathologies. In particular, we aimed to mimic the conditions of the whole route from the hAM retrieval to production chain and to the introduction of a sutureless AmnioClip-plus system to a patient [2]. The whole process implies the donation of the hAM with immediate processing followed by the first step of fresh frozen cryopreservation, after which the frozen hAM is further thawed, installed in the AmnioClip system, and then cryopreserved again until the final second thawing directly prior to use in a patient.

Among the numerous clinically relevant properties of the hAM, including the activation of epithelialization, the suppression of inflammation and scarring, the ability to stimulate the proliferation and migration of limbal stem cells, and the ability to suppress vascularization, are extremely valuable in the field of ophthalmology [1,7,9,18]. These mechanisms of action are delivered by a wide range of cytokines and growth factors contained in the hAM and released to the eye surface when applied to a patient [1,8,9]. In this work, we analyzed the hAM for levels of EGF, which is considered to be a key player in supporting the proliferation of the corneal epithelium; TGF-β1, which controls fibroblast activity during wound healing; and HGF and bFGF, which also support organ regeneration and tissue repair [9,50]. The content was evaluated in the lysates from the non-frozen hAMs, as well as in the lysates after one and two freezing cycles. While many researchers have analyzed the expression of these factors on mRNA levels [2,21,51], we specifically focused on the actual content of synthesized proteins present in the hAM. We were able to detect the above mentioned growth factors in all analyzed hAM lysates independently of the applied temperature modes, showing that the double freezing was a safe strategy to preserve the desired hAM properties. In addition, besides the content in the hAM tissue, we analyzed the capability of the hAM to release these factors, since it is an important factor for therapeutic action. As the most pronounced curative effect in the ocular surface treatment is observed within the first week of hAM application [25], we analyzed the presence of the growth factors in the media incubated for 24 hours with the hAM. We were able to detect HGF and bFGF in the conditioned medium in amounts that were statistically similar for all the studied temperature modes. Interestingly, TGF-β1 and EGF were not detected in the conditioned medium after 24 hours in any of the donors and in any of the investigated temperature conditions. While there is a possibility that these growth factors, though found as present in the hAMs, were not being released into the conditioned medium, we rather suppose that this may have been due to their degradation outside of the hAM tissue in the incubation solution within the studied 24 hour period at 37 °C. During the lysate preparation, protease and phosphatase inhibitors were applied to prevent protein degradation, whereas the incubation of hAM in phosphate buffered saline (PBS) for the generation of the conditioned medium occurred under physiological conditions without any additives. Other studies were also not able to detect certain growth factors in the conditioned medium, but high variations in the factor content depending on the processing methods were observed [41,50,52]. Thus, we can assume that therapeutic effects of amniotic TGF-β1 and EGF are either achieved within a short term action period or participate in a complex action of the whole cytokine content present in the hAM. In general, we observed higher variations in the growth factor content between the donors than between the studied temperature modes. High interdonor variation is confirmed by numerous works of other researchers and is a typical diversity for virtually any donor material in general [41,50,53,54]. 

In addition to growth factors, we analyzed the content of extracellular matrix proteins, which have a major contribution to a wound healing processes supported by the hAM in the treatment of ocular surface pathologies. In particular, we studied the presence of laminin and hyaluronic acid, which deliver mechanical support for the hAM tissue and facilitate the binding of biologically active molecules and a range of cells, thus regulating re-epithelization and cell migration [22,55,56]. We were able to detect both laminin and hyaluronic acid in hAM lysates, as well as in a conditioned medium, which has also been confirmed by other studies [55]. No significant differences were observed between the samples from the non-frozen hAM and once and two times frozen samples, while typical interdonor variations were present. 

Since the hAM is mainly used in ophthalmology as biological dressing and is applied directly to the patient’s ocular surface, it was crucial to evaluate morphological stability after the impact of one and two freezing–thawing cycles. This is especially important in the case of sutureless application systems, such as AmnioClip-plus, where structural stability is required for a proper installation and further functioning [2]. Similar to the other researchers, we observed pronounced visual variations between the morphology of the hAM from individual donors, as well as between the regions of a single hAM (data not shown) [1,41,53]. At the same time, the histological examination of HE-stained samples did not reveal significant differences in morphology within individual hAM samples after the impact of single and double freezing–thawing in comparison to the non-frozen samples. We further performed an ultrastructural analysis of the hAM with the application of SEM, since various authors have reported controversial results on the stability of the ultrastructure after cryopreservation [57,58]. However, these reported variations can be explained by the differences in cryopreservation approaches, which in turn are appointed by desired outcome and depend on numerous factors, such as the size of the samples, whether or not it is necessary to maintain cell viability after thawing, whether or not and what type of CPA is used, and which cooling and thawing rates are used [9,10]. With the application of a fresh frozen method, which is used for ophthalmological hAM preparations in clinics and was also adapted in the frames of this study, we observed the morphological stability of the basal hAM area after two freeze–thaw cycles. The maintenance of cell viability is not expected in such clinically applied hAM preparations and was therefore not evaluated in this work. In case the cellular viability is required, another cryopreservation approach utilizing CPAs and controlled cooling rates should be applied [10,59,60]. 

The behavior of extracellular and intracellular ice crystal formation, as well as the temperature of ice nucleation, are primarily important parameters to consider during freezing and thawing procedures for biological tissues in general and for the hAM in particular [10,60]. Thus, in this work, we performed cryomicroscopic observations following the same single and double freezing–thawing protocols as in the rest of the study. We aimed to find out whether these steps affect the structural integrity of the hAM and evaluated the contribution of ice formation and re-crystallization. In confirmation of the described above histological and ultrastructural analysis data, we did not observe significant changes in the structure of the hAM after single and double freezing–thawing. This was also partially described in our previous studies with cryomicroscopic observations, where strategies to preserve cellular viability in the hAM tissue after thawing were discussed [10]. 

Mechanical properties, such as elasticity and mechanical strength, are essential criteria for the primary evaluation of the hAM for suitability for clinical use. Generally, the heterogeneous and isotropic viscoelastic properties of the hAM determine the potential of its application [61,62]. The direct fixation of the hAM with sutures to the ocular surface, or sutureless fixation with prior insertion to the contact lens-like system, strongly relies on uncompromised mechanical parameters. Here, we assessed whether single and double freezing impaired the relevant mechanical properties, which could be critical for clinical therapies involving the hAM. The size of the enclosed area within the hysteresis loop represents the potential energy that could be stored between loading and unloading. This value thus indirectly stands for elasticity of the hAM. In the first cycle, the fibers of the extracellular matrix (ECM) were aligned within the tissue. This ability appears to have been affected by repeated freezing and thawing procedures because the corresponding values decreased. The tissue became more inelastic as the number of freezing cycles increased. At the same time, the samples showed a similar behavior from the second loading cycle, regardless of the number of freezing and thawing procedures. Thus, it can be assumed that studied mechanical properties of ECM fibers are not affected by the freezing and thawing procedures. Hence, repeated freezing and thawing procedures primarily affected the biomechanical behavior of the composite tissue structure and, to a lesser extent, the structural elements of the tissue (e.g., collagen or elastin fibers). 

The improvement, preservation, and maintenance of the clarity of a patient’s vision are the main goals in ophthalmology. Since treatment involving the hAM implies the direct application to the eye surface, the transparency of the hAM is among the major benefits allowing for the maintenance of eye sight throughout the therapy process [1]. This parameter is especially critical when systems like AmnioClip-plus are being used, where the hAM is applied in the format of a contact lens. We designed a simple but robust and representative test to assess the transparency of the hAM. The hAM was placed on a flat carrier with an aperture on top of a fully transparent cell culture dish with printed text of various sizes in typical book reading fonts in black on a white background underneath. Our method clearly allowed us to easily distinguish even slight variations in hAM transparency. All studied non-frozen hAMs featured enough transparency to clearly distinguish the underlying text in different font sizes. No differences were observed between the hAM of the same donor before and after evaluated freezing–thawing procedures, thus making a double freezing a safe technique in terms of the preservation of hAM clarity for use in ophthalmological therapies. 

## 4. Materials and Methods

### 4.1. Experimental Design

Placental amniotic membranes were retrieved under sterile conditions after caesarian-section delivery and were prepared according to clinical standards for application in ophthalmology (EDQM Council of Europe, 4th Edition of the Guide to the Quality and Safety of Organs for Transplantation). Afterwards, fragments of the hAM were subjected to single and double freezing cycles, and they were compared to fresh (non-frozen) samples. Growth factors and extracellular matrix proteins contents, morphology, ultrastructure, and a range of physical properties were evaluated. A cryomicroscopic analysis of possible damage occurring in the amniotic tissue due to ice crystal formation was performed. In order to explore the capability of the hAM to release clinically relevant extracellular matrix proteins and growth factors before and after the freezing procedures, tissue fragments were placed in an isotonic saline-buffered solution, followed by the analysis of the conditioned medium after 24 hours. Additionally, non-frozen hAM fragments, as well as fragments after single and double freezing cycles, were lysed with a subsequent analysis of the lysate for the presence and amounts of similar matrix proteins and growth factors in the tissues. 

### 4.2. Amniotic Membrane Preparation

Eleven human placentas were obtained with routine Caesarean section delivery from healthy women at full-term pregnancy (37–40 weeks of gestation) at the Department of Gynecology and Obstetrics at Hannover Medical School, Germany (approved by Ethical Commission of Hannover Medical School, Ethic votum No.2396-2014). The afterbirth was delivered to the laboratory within 1–2 hours in a moist chamber. All human samples were obtained in an anonymized form with the written informed consent of the patients.

The hAM was prepared under sterile conditions according to the manufacturing protocol of the German Society for Tissue Transplantation (DGFG) regulated by the guide to the quality and safety of tissues and cells for human application (EDQM—European Directorate for the Quality of Medicines). In brief, human term placenta after caesarean section was delivered to the laboratory and immediately processed. At first, the placenta was washed several times in an isotonic 0.9% NaCl solution, and then the hAM was manually detached from chorion and placed on BD Visiwipe™ Adhesive Instrument Wipes as carriers (Beaver-Visitec Int., Waltham, MA, USA). Next, the hAM was washed again in an isotonic 0.9% NaCl solution, followed by 1 hour equilibration in antibiotic solution containing 50 E/ml of penicillin and und 50 µg/ml of streptomycin, with a subsequent final wash in isotonic NaCl. After the last washing, the hAM was cut into 15 mm^2^ pieces and frozen in individual packages. For the transparency test, 100 mm^2^ samples were used. The hAM was frozen without the addition of a cryoprotective agent (fresh frozen method), with storage following at −80 °C [32,33]. The thawing of the samples was performed for 30 minutes at room temperature, followed by the second freezing step. The time interval between thawing and the second freezing was introduced to mimic the production process of the installation of the hAM into AmnioClip dual ring system for application in ophthalmology. Fresh, the non-frozen hAM served as a control.

### 4.3. Growth Factors and Extracellular Matrix Components Assessment

An ELISA was used to measure the contents of the growth factors EGF, HGF, TGF-β1, and bFGF (R&D systems, Minneapolis, MN, USA), as well as the extracellular matrix proteins laminin (Abcam, Cambridge, UK) and hyaluronic acid (R&D systems). The presence of these proteins was quantified in hAM lysates, and the capability of hAM to release these substances was measured in the conditioned medium after incubation with the hAM. 

Lysates were prepared by the mechanical shredding of hAM tissue in a RIPA buffer (Thermo Fisher Scientific, Waltham, MA, USA) in the presence of protease and phosphatase inhibitors. The conditioned medium was collected after 24 hours of incubation of 15 mm^2^ of hAM in 0.5 mL of PBS at 37 °C.

Quantitative sandwich ELISA assays were used according to the manufacturer’s protocol. In brief, a monoclonal antibody that was specific for each factor was pre-coated onto a microplate. Standards and samples were pipetted into the wells where the studied growth factor or extracellular matrix component present in the sample was bound by an immobilized antibody. After washing away the unbound substances, an enzyme-linked specific polyclonal antibody was added to the wells. Following a wash to remove the unbound antibody-enzyme reagent, a substrate solution was added to the wells. Results were acquired with a Biotek Synergy™ 2 Multi-Mode Microplate Reader microplate reader (BioTek Instruments, Inc., Winooski, VT, USA) at a wavelength of 450 nm. The quantity of the studied substances is presented as a relative to the total protein content in the sample. 

### 4.4. Morphological Evaluation 

Morphology was evaluated after HE stainings. Non-frozen, one time frozen, and two times frozen tissue samples were fixed in a 4% buffered formalin solution (Fishar, Saarbrücken, Germany). After 24 hours, the samples were embedded in paraffin and cut with a rotary microtome into 2 μm slides. The sections were deparaffinized in 100% xylene (3 times for 7 minutes) and then rehydrated in a gradient of ethanol concentrations to water (100%, 90%, 70%, 50%, and 20% ethanol followed by distilled and tap water, each for 5 minutes) and stained with Mayer´s hematoxylin and eosin (VWR International GmbH, Darmstadt, Germany). Stained sections were dehydrated in increasing ethanol concentrations (in a reverse manner to the rehydration steps described above), then moved to xylene and mounted in a DPX medium (Sigma-Aldrich, St. Louis, Missouri United States). Histological sections were documented on a Keyence Biozero microscope (Keyence Germany GmbH, Neu-Isenburg, Germany).

### 4.5. Cryomicroscopy

Cryomicroscopy was conducted according to a modified protocol that was previously established by our group [63] using an AxioVert M1m microscope (Carl Zeiss, Oberkochen, Germany) with BCS196 Linkam cryostage (Linkam Scientific Instruments, Tadworth, UK) to observe the morphological changes and integrity of the hAM upon two freeze–thaw cycles. The hAMs were cut with a 10 mm puncher, washed twice with PBS (Carl Roth, Karlsruhe, Germany), fixed between two 0.17 mm cover slips, placed onto a quartz crucible (Resultec Analytic Equipment, Illerkirchberg, Germany), and transferred to a temperature-controlled silver block of the cryostage mounted on the microscope. The samples were brought to 4 °C at a 10 K/min cooling rate and allowed to equilibrate at 4 °C for 2 min (Supplementary Figure S1). The first freeze–thaw cycle included freezing at 5 K/min down to −80 °C, holding at −80 °C for 2 min, and thawing back to 4 °C with 100 K/min. Before the second freeze–thaw cycle of the same hAM, according to the above mentioned protocol, the samples were allowed to equilibrate at 4 °C for 2 min. A Retiga EXi high speed camera (Qimaging, Surrey, BC, Canada) was used to record the images with a frame rate of 6 fps. The cryostage was controlled using the Linksys32 software (Linkam Scientific Instruments, Tadworth, UK).

### 4.6. Scanning Electron Microscopy

SEM was used to observe the ultrastructural morphology of the hAM, according to the previously established protocol with modifications [64]. For these purposes, the non-frozen and frozen hAM (1× and 2× freeze–thaw cycles) were first washed two times with a 0.1 M cacodylate buffer (CAC, pH 7.4, Carl Roth, Karlsruhe, Germany) for 10 min and fixed with 2.5% glutaraldehyde (Carl Roth) in 0.1 M CAC for 90 min. The secondary fixation of the samples was done using 1% osmium tetroxide in distilled water (Carl Roth, Karlsruhe, Germany) for 90 min. After post-fixation, the samples were washed twice with distilled water (10 min each wash) and dehydrated in a series of ethanol concentrations (25%, 50%, 75%, 90%, and 100%) for 20 min for each step. After dehydration, the samples were exposed to chemical drying using hexamethyldisilazane (HMDS, Sigma Aldrich, Darmstadt, Germany). The first drying step included a 1:1 ratio of ethanol to HMDS for 10 min following two last drying steps in 100% HMDS for 10 min. After being air dried overnight, the samples were fixed on SEM stubs using conductive double-sided glue tape, sputter coated with gold–palladium for 30 s, and visualized at a 15 kV accelerating high voltage and a 7 mm working distance in a high vacuum using a scanning electron microscope (S3400N, Hitachi, Tokyo, Japan). 

### 4.7. Mechanical Test

The mechanical properties of the hAM were evaluated by performing a dynamic uniaxial tensile test. BD Visiwipe carriers (10 × 30 mm) were mounted in a custom-made clamping system fabricated with polylactic acid (PLA, German RepRap GmbH, Feldkirchen, Germany) via 3D-printing (Ultimaker 3 extended, Utrecht, Netherlands). The clamping system ensured the protection of the samples during freezing–thawing, as well as guaranteeing a defined gauge length of 10 mm. The underlying BD Visiwipe carrier was cut laterally in half before the tensile testing was performed. Samples were tested fresh, as well as after 1 and 2 freeze–thaw cycles (*n* = 3 for each condition). Freezing and storage (24 hours) was performed in a −80 °C refrigerator (Sanyo, Osaka, Japan). Samples were thawed by gently rinsing in a phosphate buffer solution at 37 °C. The mechanical test was performed with a tensile testing machine (Electroforce LM1 test bench, TA Instruments, Milford, MA, USA) according to the test protocol shown in Supplementary Figure S2. Briefly, the samples were preloaded with 10 mm/min to 5% elongation followed by five sinusoidal loading and unloading cycles (1Hz) from 5% to a maximum of 15 % elongation. Force and elongation were recorded and used to analyze the hysteresis of each loading cycle by using custom-made SciLab software, as previously described by our group [65].

### 4.8. Transparency Test

In order to evaluate the transparency of the hAM before and after freezing–thawing cycles, 10 mm^2^ of non-frozen, one-time frozen, and two times frozen hAMs were placed on BD Visiwipe carriers with an aperture of 15 × 30 mm over text of varied font sizes (10–16 point) in transparent 100 mm cell culture dishes. The clarity of the text visibility was visually assessed under the day light conditions. 

### 4.9. Data Analysis and Statistics

The data were obtained from 11 independent hAM donors, with individual experiments performed at least in quadruplicate (*n* ≥ 4). Statistically significant conclusions were acquired with an analysis of mean values and standard deviation calculations (mean ± SD) with the Mann–Whitney test and Fisher’s method. Parametric changes were considered statistically significant at *p* < 0.05. Past V. 3.15 Software (UiO, Norway) and GraphPad Prism 5.02 (GraphPad Software Inc., San Diego, CA, USA) were used for statistical calculations and data analysis.

## 5. Conclusions

The presence of HGF, bFGF, EGF, and TGF-β1 growth factors, as well as laminin and hyaluronic extracellular matrix proteins acid, were detected in all analyzed hAMs. Pronounced variations of the content of the studied substances were observed between the donors. Virtually no changes in the contents of the studied growth factors and extracellular matrix proteins were detected after double freezing with the described fresh freezing method used for clinical application. Morphology, ultrastructure, and a range of mechanical properties were not altered significantly after two freeze–thaw cycles. Therefore, the hAM can be frozen and thawed at least two times without significant changes to the studied clinically relevant features necessary for application in regenerative ophthalmology. At the same time, primary interdonor variations should be considered. The development of the strategy for the selection of amniotic membranes with specific properties depending on the aim of intended clinical application and the nature of treated pathology should be deliberated. An alternative could be the pooling of several hAM extracts from different preparations to allow for further forms of application.

## Supplementary Materials

Supplementary materials can be found at https://www.mdpi.com/1422-0067/21/11/4029/s1. Figure S1: Freeze–thaw protocol for cryomicroscopic observations of one-time (1×) and two times (2×) frozen hAM, Figure S2: Test protocol for the mechanical characterization of hAM. Samples were preloaded to 5 % elongation before performing five sinusoidal loading and unloading cycles from 5 % to 15 % elongation. The graph displays signals of the non-frozen, one-time (1x) and two times (2x) frozen hAM, as well as signal of the machine motor itself.
