# Supplementary material for: Repeated Freezing Procedures Preserve Structural and Functional Properties of Amniotic Membrane for Application in Ophthalmology
Source: Int J Mol Sci. 2020 Jun 4;21(11):4029. doi: 10.3390/ijms21114029 (PMC7312941; doi:10.3390/ijms21114029)
Supplement: Supplementary file 1 [file ijms-21-04029-s001.pdf]

Supplementary Materials

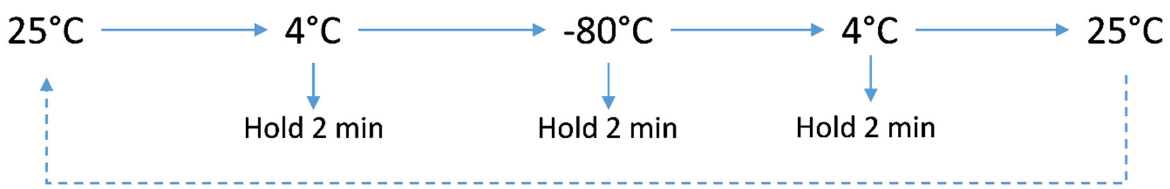

Figure S1. Freeze-thaw protocol for cryomicroscopic observations of one-time (1×) and two times (2×) frozen hAM.

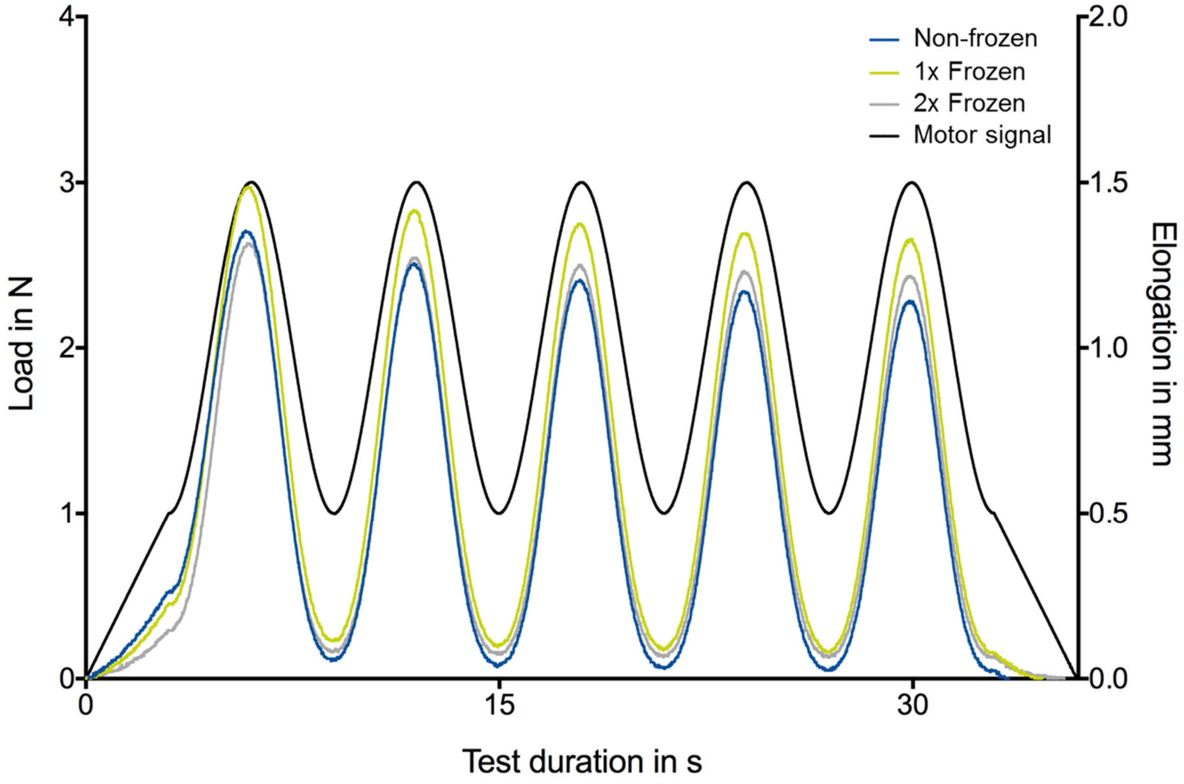

Figure S2. Test protocol for the mechanical characterization of hAM. Samples were preloaded to 5 % elongation before performing five sinusoidal loading and unloading cycles from 5 % to 15 % elongation. The graph displays signals of the non-frozen, one-time (1×) and two times (2×) frozen hAM, as well as signal of the machine motor itself.
